# Enhanced Wear and Corrosion Resistance of AlCoCrFeNiMoTi High-Entropy Alloy via B Addition by Laser Cladding

**DOI:** 10.3390/ma18204651

**Published:** 2025-10-10

**Authors:** Sansan Ao, Jiaxun Sun, Ziyuan Qi, Youxiang Wei, Hongyu Chen, Yang Li

**Affiliations:** 1 School of Materials Science and Engineering, Tianjin University, Tianjin 300350, China; ao33@tju.edu.cn (S.A.); 18260733197@163.com (J.S.); tjuqizy@163.com (Z.Q.); 3021001828@tju.edu.cn (Y.W.); chy0123@tju.edu.cn (H.C.); 2 The National Key Laboratory of Particle Transport and Separation Technology, Tianjin 300350, China

**Keywords:** laser cladding, high-entropy alloy, in situ TiB_2_, friction–wear behaviors, corrosion resistance

## Abstract

To address the synergistic degradation mechanisms in engineering service environments, we propose a boron microalloying strategy to enhance the multifunctional surface performance of AlCoCrFeNiMo-based high-entropy alloys. AlCoCrFeNiMoTiBx coatings (x = 0, 0.5, 1, and 1.5) were fabricated on Q235 steel substrates using laser cladding. The microstructure of the coatings was characterized using scanning electron microscope (SEM) and energy dispersive spectrometer (EDS), while their wear and corrosion resistance were evaluated through tribological and electrochemical tests. The key findings indicate that boron addition preserves the original body-centered cubic (BCC) and σ phases in the coating while promoting the in situ formation of TiB_2_, leading to lattice distortion. With increasing B content, the BCC phase becomes refined, and both the fraction and size of TiB_2_ particles increase. Boron incorporation improves the coating’s microhardness and wear resistance, with the highest wear resistance achieved at x = 1, where abrasive and oxidative wear predominate. At lower content (x = 0.5), B enhances the stability of the passive film and thereby improves corrosion resistance. In contrast, excessive formation of large TiB_2_ particles introduces defects into the passive film, accelerating its degradation.

## 1. Introduction

The concept of high-entropy alloys (HEAs), based on multi-component design and atomic-level homogeneous mixing, has emerged as a prominent research focus in materials science since its inception [1,2]. High configurational entropy within the HEA (ΔS > 1.5 R, where R denotes the gas constant) favors the formation of single-phase or two-phase solid solutions [3,4]. Their unique features-including high entropy, lattice distortion, sluggish diffusion, and the cocktail effect-form the theoretical basis for designing alloy compositions tailored to specific performance requirements [5,6,7]. However, conventional HEAs are typically rich in high-cost elements, which limits their large-scale production and processing [8,9]. Nevertheless, HEAs exhibit exceptional mechanical properties, high hardness, excellent high-temperature oxidation resistance, and superior corrosion resistance, making them promising candidates for coating applications [10,11,12]. Among the various techniques for fabricating HEA coatings, laser cladding has gained prominence owing to its advantages of a narrow heat-affected zone, low dilution, minimal deformation, and strong metallurgical bonding with the substrate [13]. Owing to these benefits, laser cladding is widely recognized in the scientific community as the optimal technique for preparing HEA coatings [2,8,9].

In the early stage of HEA coating development, the single-phase face-centered cubic (FCC) CoCrFeNi system was widely used, but it was criticized for its insufficient hardness and poor wear resistance [14,15]. To address this issue, researchers introduced Al in varying amounts, enabling the transformation from an FCC structure to an FCC + body-centered cubic (BCC) dual-phase structure and ultimately to a single-phase BCC structure [16]. Currently, AlCoCrFeNi, a representative single-phase BCC HEA, is widely applied. With advancements in materials science and increasing demands for extreme environments, the development of highly wear-resistant and corrosion-resistant materials has become imperative. The introduction of solid solution strengthening elements (e.g., Ti, Mo, Nb) has proven to be an effective strategy for enhancing the properties of AlCoCrFeNi HEAs [17]. For instance, increasing the Ti content alters the phase composition of Al_0.45_CoCrFeNiTi_x_ HEA, leading to a transition from a BCC + FCC structure to a BCC + FCC + L21 (Ordered FCC phase) structure, where the induced solid solution strengthening effect significantly improves microhardness [18]. Similarly, Nb addition in Al_0.5_CoCrFeNi HEAs promotes the formation of Laves phases, refining the solidification rate and phase transformation process while restricting FCC phase growth [19]. Mo not only introduces lattice distortion and enhances solid solution strengthening effects, thereby improving coating strength and wear resistance [20], but also increases the pitting resistance of the passive film [21]. These improvements make Mo a promising element for enhancing HEA coatings in extreme environments.

Inspired by the design concept of in situ ceramic phase-reinforced metal matrix composites [22], researchers have attempted to incorporate hard particles (e.g., TiC, Al_2_O_3_, SiC, and TiB_2_) into HEAs to endow the metallic matrix with not only high strength and wear resistance but also good ductility and plasticity, thereby achieving superior comprehensive mechanical properties [23]. Numerous intriguing studies have emerged in this field. For example, FeMnCrNiCo + x(TiC) coatings were fabricated on 4Cr5MoSiV1 die steel, revealing that a TiC content of 5 wt.% significantly enhanced coating strength, while excessive doping (10 wt.%) led to brittle precipitates that induced crack formation [24]. Among these ceramic phases, TiB_2_ is regarded as an outstanding reinforcement owing to its high melting point, high hardness, strong interfacial bonding with the matrix, and excellent chemical stability. Its role in enhancing hardness and wear resistance has been well established. For instance, the addition of 1 at.% TiB_2_ to an Al_1.5_Co_0.5_CrFeNi_2_ HEA coating increased its microhardness to 939.37 HV while reducing the wear rate to 1.13 × 10^5^ mm^3^/(n·m) [25]. Moreover, in (Fe_50_Mn_30_Co_10_Cr_10_)_0.8−x_(TiB_2_)_x_Mo_0.2_ (x = 0, 0.05, 0.1, and 0.2) HEA coatings, an appropriate amount of TiB_2_ not only enhanced wear resistance but also provided stable support for the passive film. Additionally, TiB_2_ suppressed the FCC-to- hexagonal close-packed (HCP) phase transition and promoted the formation of the σ phase in the coating [14]. These findings suggest that the rational addition of TiB_2_ could facilitate the development of HEA coatings with high hardness, excellent wear resistance, and superior corrosion resistance.

We selected AlCoCrFeNiMoTi HEA as the base system and enhanced the coating’s wear resistance and corrosion resistance through the addition of boron. Specifically, we employed laser cladding technology to deposit AlCoCrFeNiMoTiB_x_ (x = 0, 0.5, 1, 1.5) HEA coatings onto low-carbon steel substrates. Through microstructural characterization, we investigated the mechanism by which boron influences microstructure and analyzed the intrinsic mechanisms by which boron enhances wear resistance and corrosion resistance, aiming to provide a theoretical foundation for the preparation of high-performance coatings.

## 2. Materials and Experimental Works

This study used high-purity gas-atomized Al, Co, Cr, Ni, Ti, and Mo powders (45–105 μm) in an equimolar ratio as raw materials. TiB_2_ was in situ formed by adding varying B amounts, defining coatings as S1 (x = 0), S2 (x = 0.5), S3 (x = 1), and S4 (x = 1.5). The powders were weighed according to the designed composition and thoroughly blended using a V-type mixer (V-10, Xinyang Equipment Technology Co., Ltd., Wuxi, China). Q235 steel samples with dimensions of 80 × 80 × 10 mm^3^ served as the substrate. After grinding, polishing and drying the substrate was pre-coated with powder to a thickness of approximately 1 mm. The HEA coating was fabricated using a fiber laser (IPG-YLS-10000, IPG Photonics Corporation, Beijing, China) with the following optimized parameters: power 800 W, scanning speed 12 mm/min, overlap ratio 30%, and defocus 15 mm.

The coated samples underwent cutting, grinding, and polishing before characterization. The phase composition of the coating was determined by X-ray diffraction (XRD, Ultima IV, Shanghai Lijing Scientific Instrument Co., Ltd., Shanghai, China) under the following conditions: tube voltage 40 kV, tube current 40 mA, and scanning speed 4°/min. Microstructure and elemental distribution were examined using scanning electron microscopy (SEM, JSM-7800F, JEOL Ltd., Beijing, China) and energy dispersive spectroscopy (EDS, JEOL Ltd., Beijing, China). The sample surfaces were ground and subsequently polished to minimize the influence of surface conditions on the test results. Vickers hardness (HV-1000A, Laizhou Huaxing Testing Instrument Co., Ltd., Yantai, China) was measured, and wear resistance was tested using a friction and wear tester (HT-1000, Zhongke Kaihua Technology Development Co., Ltd., Lanzhou, China) at room temperature, with a YG6 ball under a 20 N load for 30 min. SEM and EDS characterized the wear morphology.

After grinding and polishing, the specimen was rinsed with anhydrous ethanol. Corrosion resistance in 3.5 wt.% NaCl was evaluated via electrochemical tests using a three-electrode system (Gamry Interface 1000, Gamry Electrochemistry, Shanghai, China). The coating serves as the working electrode, with an area of 1 cm^2^. Platinum acts as the auxiliary electrode, while the saturated calomel electrode functions as the reference electrode. Electrochemical impedance spectroscopy (EIS) was performed from 10^5^ Hz to 10^−2^ Hz at a 10 mV amplitude, while potentiodynamic polarization curves were recorded from −0.5 to 1.5 V (versus open circuit potential, vs. OCP) at 0.5 mV/s.

## 3. Results and Discussion

### 3.1. Microstructure Characterization of HEA Coatings

**Figure 1:**
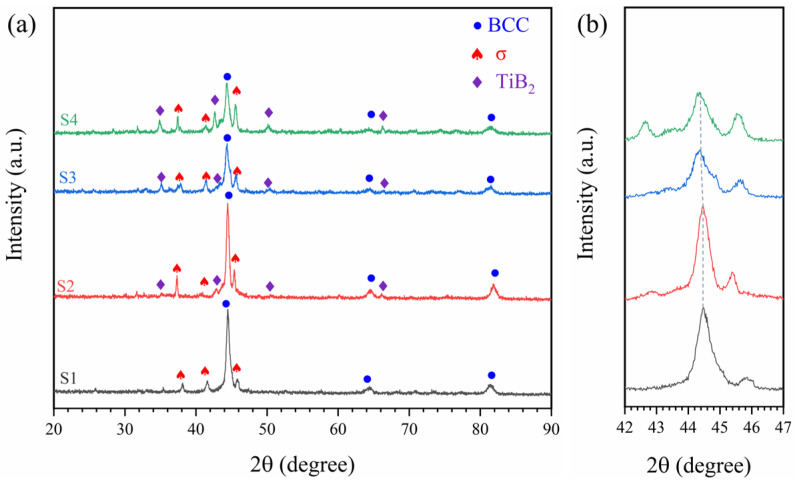
(**a**) XRD results of AlCoCrFeNiMoTiBx HEA coating, and (**b**) local magnification near the main peak of 42–47°.

XRD results (Figure 1) show diffraction peaks at 44.5°, 64.8°, and 82.0°, corresponding to the (110), (200), and (211) planes of the BCC structure. Alongside the BCC phase, all coatings contain a σ phase. B addition did not change the phase composition but induced the in situ formation of TiB_2_ in the molten pool, with its diffraction intensity increasing at higher B levels. Moreover, the main BCC peaks exhibited noticeable shifts, with the most significant shift toward lower angles occurring as B content increased from 0.5 to 1, indicating pronounced lattice distortion [26].

**Figure 2:**
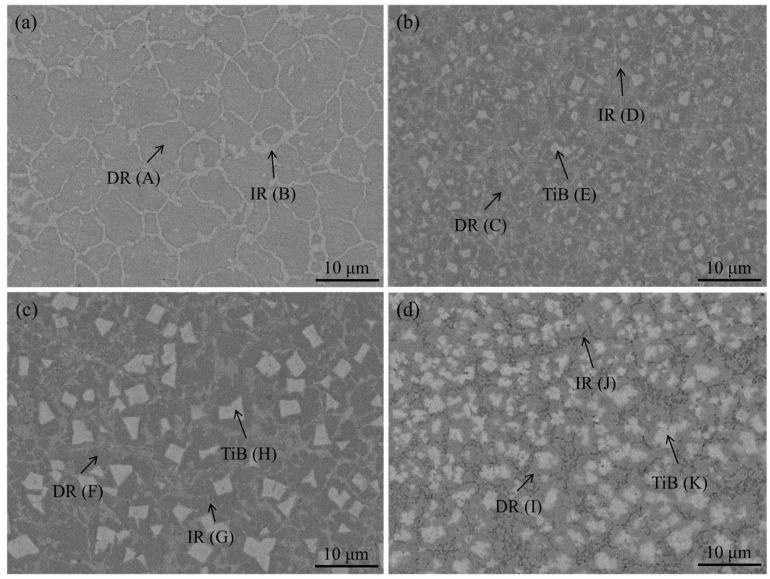
SEM BSE diagram of AlCoCrFeNiMoTiBx HEA coating: (**a**) S1, (**b**) S2, (**c**) S3, and (**d**) S4.

Figure 2 shows the microstructural features of the AlCoCrFeNiMoTiBx HEA coatings. All coatings exhibit a typical dendritic (DR)-interdendritic (IR) morphology. The DR region is enriched in Al, Cr, and Fe, whereas the IR region contains higher concentrations of Ni, Ti, and Mo (Table 1), with Mo-rich σ phase distributed within the IR region. According to classical solidification theory, high-melting-point elements segregate into the primary solidification region (DR), while low-melting-point elements preferentially accumulate in the final solidification region (IR) [1,27]. Additionally, elements with larger atomic radii are pushed into the IR region during diffusion, ultimately forming this DR–IR microstructural feature.

When boron is introduced into the coating, the strong negative mixing enthalpy between Ti and B (−58 kJ/mol) promotes atomic bonding and facilitates the in situ formation of TiB_2_, which is dispersed throughout the DR region. At x = 0.5 and 1, TiB_2_ exhibits a block-like morphology, whereas excessive B content drives a transition from block-like to cellular structures. With increasing B content, both the size and volume fraction of TiB_2_ increase.

**Figure 3:**
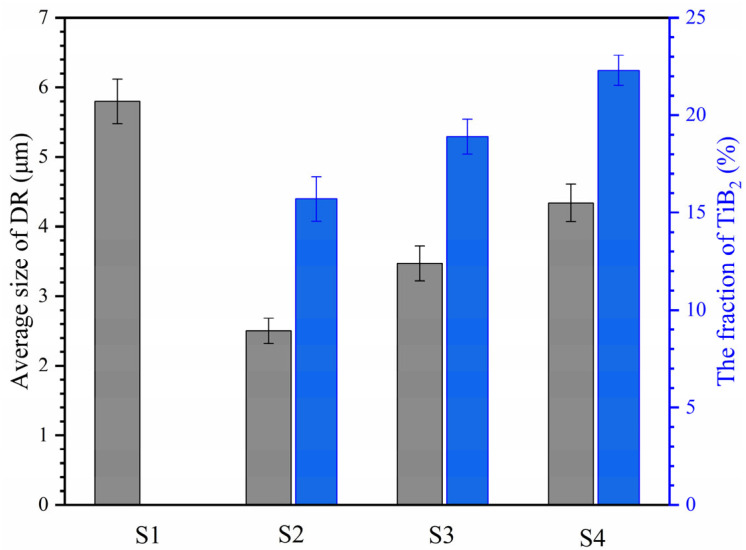
Statistical histogram of AlCoCrFeNiMoTiBx HEA coating DR Region average size and TiB_2_ proportion.

During laser cladding, high-melting-point TiB_2_ precipitates in situ during the solidification of the molten pool and subsequently enhances laser absorption, leading to localized high-temperature regions. TiB_2_ particles remain suspended in the liquid metal, and with continued laser exposure, the molten pool stabilizes in terms of size and shape. The temperature gradient induces surface tension-driven convection (Marangoni effect) [28], causing liquid metal to flow from the center of the molten pool towards the surrounding cooler regions. TiB_2_ provides nucleation sites for new crystal growth, reducing local undercooling and thereby inhibiting grain growth [29,30]. Consequently, the addition of B refines the BCC phase, with the BCC phase size in S2 decreasing by 56.9% compared to S1 (Figure 3). However, as the B content further increases, TiB_2_ particles collide, aggregate, and grow due to convection, leading to an increase in both content and volume fraction. When excessive B is added, TiB_2_ struggles to maintain its block-like morphology and evolves into a flocculent cellular structure according to the principle of minimum surface energy. At this stage, the aggregated and coarsened TiB_2_ particles can no longer effectively act as nucleation sites, resulting in an overall increase in the phase size within the DR region.

### 3.2. Microhardness and Room Temperature Wear Resistance Analysis of HEA Coatings

**Figure 4:**
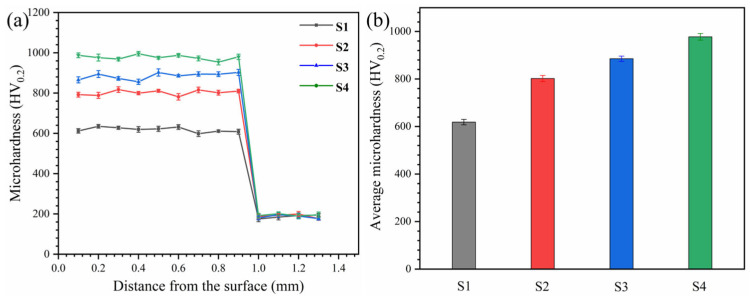
(**a**) Microhardness distribution of AlCoCrFeNiMoTiBx HEA coating, and (**b**) statistical diagram of average hardness of coating.

The microhardness distribution of AlCoCrFeNiMoTiBx HEA coatings is shown in Figure 4. The hardness of all coatings significantly exceeds that of the Q235 matrix (approximately 186.7 HV). As B content increases, the microhardness of the coatings also increases, with S1, S2, S3, and S4 exhibiting hardness improvements of 29.7%, 43.1%, and 58.0%, respectively.

The improvement of microhardness is mainly attributed to the following aspects: (i) First is the substantial increase in the content of high-hardness TiB_2_, which plays a supporting role and effectively prevents dislocation movement, causing dislocation entanglement [31]. (ii) Grain refinement induces fine grain strengthening [32]. According to the Hall-Patch theory, fine grains contribute to the improvement of hardness and strength. (iii) Obvious lattice distortion occurs in the coating, resulting in solid solution strengthening effect and improving the hardness of the coating.

**Figure 5:**
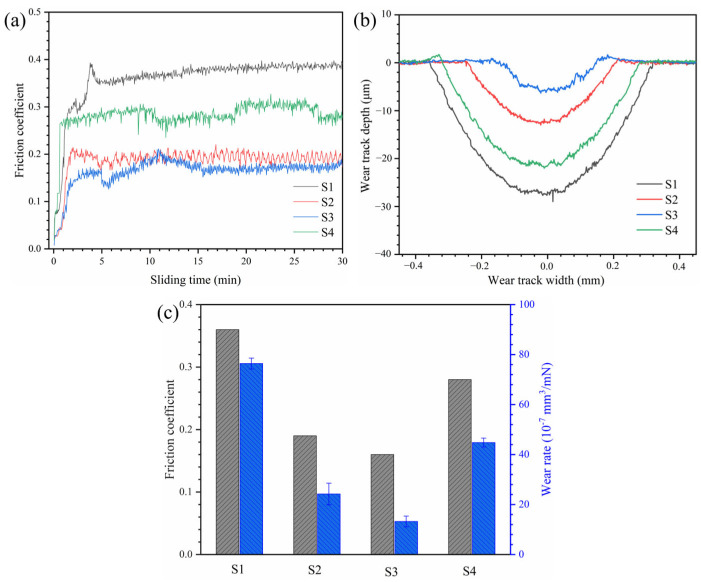
(**a**) The change curve of friction coefficients of AlCoCrFeNiMoTiBx HEA coating over time (**b**) the two-dimensional wear morphology of AlCoCrFeNiMoTiBx HEA coating, and (**c**) the statistical diagram of the average COF value and wear rate of each coating.

As shown in Figure 5a, the friction coefficient (COF) of the AlCoCrFeNiMoTiBx HEA coatings initially increases rapidly during the early wear stage before stabilizing. The average COF values for the S1–S4 coatings were 0.36, 0.19, 0.16, and 0.28, respectively. The wear rate of each coating was calculated using Archard’s wear law, with the results presented in Figure 5c. The introduction of B effectively reduces the wear rate to varying extents. When x = 1, the wear rate reaches its lowest value, which is only 1/5.8, 1/1.8, and 1/3.4 of the wear rates of S1, S2, and S4, respectively. Similarly, both the wear width and wear depth first decrease and then increase with increasing x.

**Figure 6:**
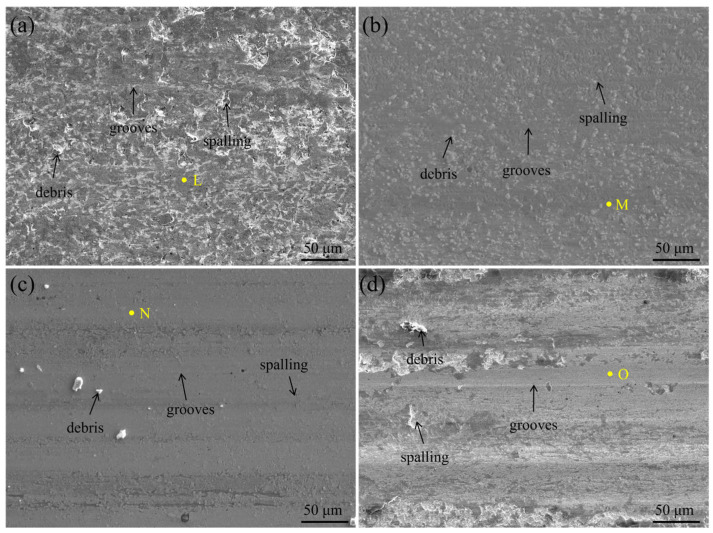
SEM images of wear morphology of AlCoCrFeNiMoTiB_x_ HEA coating: (**a**) S1, (**b**) S2, (**c**) S3, and (**d**) S4.

A detailed wear mechanism analysis was conducted based on the worn surface morphology and EDS results. As shown in Figure 6a, the worn surface of S1 exhibits significant spalling, debris accumulation, and furrows parallel to the sliding direction, indicating a rough surface with pronounced plastic deformation. Furthermore, a high concentration of oxygen was detected on the worn surface (Table 2), suggesting that repeated frictional heating during wear promotes oxidation of the coating.

The addition of B reduces the extent of adhesive wear to varying degrees. Compared to S1, the worn surfaces of S2, S3, and S4 appear smoother and more uniform.

To some extent, COF can reflect the state of the wear process. This initial increase in COF reflects the expansion of the contact area as wear progresses. The COF value of S4 fluctuates over time, which is attributed to the detachment of large-sized TiB_2_ particles [33].

The oxide on the surface of the S1 coating forms oxide debris under the action of vertical loads and shear forces of sliding motion, leading to spalling (severe adhesive wear) and further intensifying plastic deformation [34,35]. Thus, the primary wear mechanisms of S1 include abrasive wear, adhesive wear, fatigue wear, and oxidative wear.

As x increases, the proportion of in situ TiB_2_ and the hardness of the coating increase, leading to a reduction in fatigue wear. However, excessive B doping results in agglomerated TiB_2_ particles with weak bonding to the substrate. Under dynamic loading, these particles detach, re-enter the wear process, and become embedded in the worn surface. Their subsequent detachment induces the formation of large spalling pits, thereby exacerbating adhesive wear.

Based on the wear rate and worn surface analysis, the addition of boron clearly improves the wear resistance of the coatings, with the S3 coating demonstrating the best performance. The presence of TiB_2_ is the key factor underlying this enhancement. Owing to its high microhardness and low friction coefficient, TiB_2_ provides structural support to the coating, suppresses plastic deformation of the softer matrix, and improves the overall shear resistance [36]. Moreover, TiB_2_ exhibits solid lubrication behavior during wear, which reduces the coefficient of friction (COF) and further enhances wear resistance [37].

The size and proportion of TiB_2_ play a decisive role in the wear process. Relatively fine TiB_2_ particles (as in S2 and S3) have a high specific surface area, which promotes dislocation entanglement during plastic deformation, forming a hard-phase network that enhances wear resistance [38]. However, large TiB_2_ aggregates can cause stress concentration, leading to spalling (as observed in S4) and compromising the structural integrity of the worn surface. Although the grain size in S2 is smaller than that in S3, an adequate proportion of hard particles is also a crucial factor for superior wear resistance.

The phase size is another key factor influencing wear resistance. Phase boundaries typically act as barriers to dislocation motion, meaning that alloys with finer phase structures exhibit greater resistance to plastic deformation [33]. The refined dendritic region (DR) promotes the uniform distribution of the hard σ-phase, which, in synergy with TiB_2_, further enhances the alloy’s wear resistance.

Oxygen was detected on the worn surfaces of all four coatings, suggesting that the stability of the oxide film directly influences the extent of adhesive wear. The high hardness of the HEA coating minimizes oxide layer wear, allowing it to thicken over time. A thicker oxide layer reduces the frictional contact area, thereby enhancing wear resistance [39]. As a result of these combined wear mechanisms, the S2 and S3 coatings demonstrate superior wear resistance compared to the other samples.

### 3.3. Corrosion Behavior of HEA Coating

**Figure 7:**
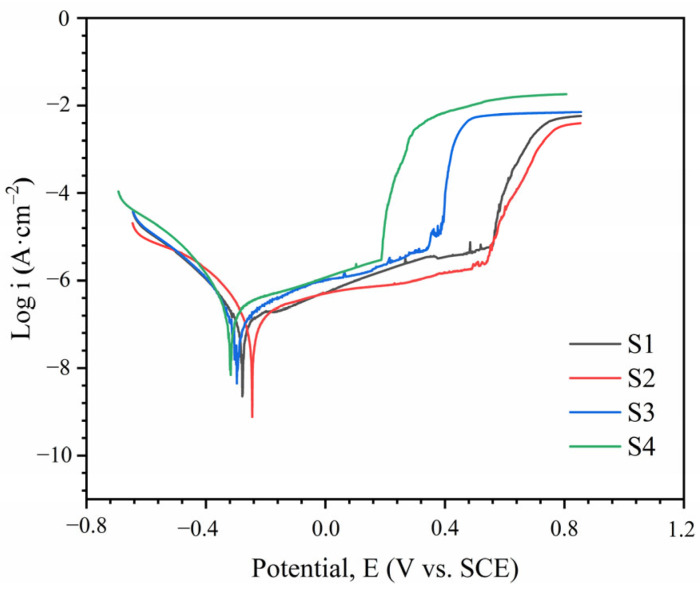
Potentiodynamic polarization curve of AlCoCrFeNiMoTiBx HEA coating in 3.5 wt.% solution.

The potentiodynamic polarization curves of AlCoCrFeNiMoTiB_x_ HEA coatings in a 3.5 wt.% NaCl solution are shown in Figure 7. All samples exhibit a passivation region, where the formation of the passive film prevents material exchange between the environment and the metal surface. Within this region, the current density increases at a reduced rate as the voltage rises. The polarization curves were fitted using the Tafel method, and the results for corrosion potential (E_corr_) and corrosion current density (i_corr_) are summarized in Table 3. E_corr_ is a kinetic parameter that evaluates the corrosion sensitivity of metal materials, while i_corr_ is commonly used to characterize the metal’s active dissolution and uniform corrosion rate [40,41]. S2 and S4 have the most positive and most negative E_corr_ values, respectively, indicating the lowest and highest corrosion tendencies. When a small amount of B is added, the coating’s corrosion resistance improves, as evidenced by a 51.0% reduction in the i_corr_ value of S2 compared to S1. However, when x ≥ 1, the corrosion resistance of the coatings decreases compared to those without B addition.

The passivation properties of a metal determine its corrosion resistance, which can be evaluated by the passivation current density (i_p_) [5]. The trend of i_p_ values is consistent with that of i_corr_, indicating that trace amounts of B enhance the coating’s passivation tendency. The relatively short passivation intervals (E_b_ − E_p_) of S3 and S4 suggest that excessive B content leads to poor passive film stability.

**Figure 8:**
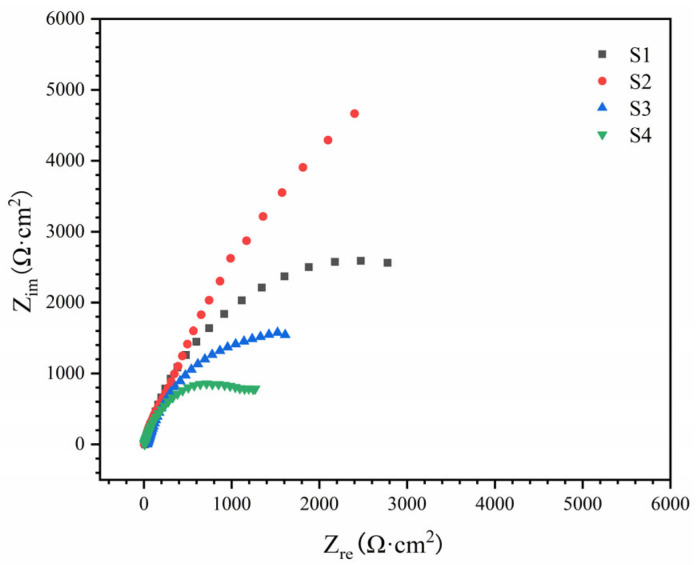
EIS Nyquist diagram of AlCoCrFeNiMoTiB_x_ HEA coating in 3.5 wt.% solution.

EIS was employed to further analyze the passivation film characteristics and corrosion resistance of the coatings, and the corresponding Nyquist plots are presented in Figure 8. All coatings exhibit capacitive arcs located in the first quadrant, with S2 showing the largest arc radius. After testing various equivalent circuits, the R_s_(Q_f_(R_f_(Q_dl_R_ct_))) model was determined to provide the best fit in this study. Here, R_s_, R_f_, and R_ct_ represent the solution resistance, passivation film resistance, and charge transfer resistance, respectively, while Q_f_ and Q_dl_ correspond to constant phase elements. The EIS fitting results are summarized in Table 4. It is evident that S2 possesses the highest values of both R_f_ and R_ct_, which is consistent with the polarization curve results, thereby further validating the accuracy of the corrosion mechanism analysis.

At the initial stage of corrosion, phase boundaries are preferentially corroded due to the presence of numerous dislocations and other defects, serving as nucleation sites for passive film formation. This promotes the rapid nucleation and growth of the passive film at the phase boundaries. Meanwhile, fine ceramic particles help reduce current density, facilitate passive film formation, and stabilize the film [42]. Dispersed ceramic particles can interact with oxides formed in the matrix phase and partially mitigate the dissolution of the matrix [43]. These factors are crucial in enhancing corrosion resistance with the addition of trace amounts of B.

However, excessive borides embedded in the passive film introduce defects and compromise its density, impairing its self-healing ability. This is reflected in the higher ip values and smaller passivation intervals observed in Figure 7. Once the passive film dissolves, galvanic corrosion, dominated by TiB_2_ and Mo-rich σ phases acting as cathodes, becomes the primary corrosion mechanism. The smaller the unit micro-cathode area, the lower the galvanic current. Clearly, this is also a key factor in the deterioration of corrosion resistance caused by excessive B addition [43,44].

In summary, regarding wear resistance, we compared the coatings obtained in this study with those exhibiting outstanding wear resistance, such as CoCrFeNi + x (NbC) coating (0.17 × 10^−4^ mm^3^/Nm) [45], WC/CeO_2_-Fe coating (3.52 × 10^−6^ mm^3^/Nm) [46], and Fe57Cr15Mo8P10C7B3 amorphous alloy coating (1.67 × 10^−5^ mm^3^/Nm) [47]. Our findings indicate that the coating we prepared exhibits a lower wear rate. Regarding corrosion resistance, our coatings also outperformed FeCrNiCoAl coating (9.8 μA·cm^−2^) [48], NiFeSiBPNb coatings (2.08 μA·cm^−2^) [49], and Fe-VC coatings (2.4 μA·cm^−2^) [50]. This demonstrates that the reinforcement strategy proposed in this study achieves breakthroughs in both wear resistance and corrosion resistance. Compared with established coatings, it proves the engineering safety and reliability of the coating.

## 4. Conclusions

This study fabricated AlCoCrFeNiMoTiBx HEA coatings on Q235 steel using laser cladding. By varying the amount of B added, the microstructure and mechanical properties of the coatings were enhanced. The key findings are as follows:

(1) The coatings contain both BCC and σ phases. The addition of B did not alter the base phases in the coating, but it reacted with Ti in the molten pool to form TiB_2_ in situ. As B content increased, the BCC phase was refined, and both the proportion and size of TiB_2_ increased.

(2) B addition improved the microhardness and wear resistance of the coatings. Microhardness increased with B content, and the coating with x = 1 exhibited the best wear resistance, attributed to the combination of a sufficient amount of small-sized TiB_2_ and solid solution strengthening. Excessive TiB_2_, especially large-sized particles, tends to spall during wear, exacerbating adhesive wear.

(3) Compared to sample S1, the i_corr_ value of sample S2 decreased by 51.0%, while R_f_ and R_ct_ increased by 23.3% and 49.2%, respectively. The improvement in corrosion resistance is attributed to the formation of a stable passivation film. However, excessive TiB_2_ acts as a defect in the passivation film, leading to premature pitting and reducing corrosion resistance.

This study demonstrates that the proposed alloy design delivers substantial improvements in both wear and corrosion resistance. The work clarifies how boron doping modifies the microstructure, establishes the linkage between microstructural features and performance, and derives general design principles for engineering coatings. We define this strategy as a generalized methodology for coating performance enhancement. While the present study does not address specific service environments, machinability, or long-term stability, we contend that with its robust performance foundation, the coating can be further optimized through compositional tuning and post-processing to enable reliable service across a broad range of applications.

## Figures and Tables

**Table 1:** Chemical composition of the AlCoCrFeNiMoTiBx HEA coatings for different areas marked in Figure 2 (at.%).

Point	Al	Co	Cr	Fe	Ni	Ti	Mo	B
A	12.4	9.3	11.6	37.8	9.7	10.5	8.7	–
B	10.1	8.5	9.4	33.4	13.1	13.2	12.3	–
C	12.5	11.7	12.5	34.9	10.5	8.9	9.0	–
D	11.3	10.3	10.1	29.7	14.3	11.4	12.9	–
E	3.4	1.8	2.1	1.3	2.5	31.0	6.4	51.5
F	11.4	9.8	11.2	40.2	7.6	11.7	8.1	–
G	9.2	9.7	10.1	34.5	11.9	12.1	12.5	–
H	2.7	0.9	3.7	3.4	1.9	33.6	4.8	49.0
I	9.6	9.3	11.2	38.9	10.5	9.4	11.1	–
J	8.7	8.1	10.5	35.7	12.3	10.1	14.6	–
K	1.8	1.3	3.7	2.1	2.4	38.9	3.6	46.2

**Table 2:** Chemical composition of the AlCoCrFeNiMoTiB_x_ HEA coatings for different areas marked in Figure 6 (at.%).

Point	Al	Co	Cr	Fe	Ni	Ti	Mo	B	O
L	9.6	7.2	8.8	25.8	5.5	9.2	9.4	–	24.3
M	6.5	4.8	10.1	27.1	6.9	7.8	5.0	3.2	28.6
N	8.9	8.1	6.8	22.4	7.2	11.2	6.8	7.2	21.4
O	6.7	7.6	7.6	19.7	6.3	7.5	11.2	9.7	23.7

**Table 3:** Electrochemical parameters of AlCoCrFeNiMoTiB_x_ HEA coatings in 3.5 wt.% NaCl solution.

Sample	E_corr_ (mV vs. SCE)	i_corr_ (μA·cm^−2^)	E_p_ (mV vs. SCE)	E_b_ (mV vs. SCE)
S1	–278	14.5	–153	542
S2	–245	7.1	–132	537
S3	–298	35.2	–104	345
S4	–317	54.1	–198	185

**Table 4:** EIS fitting results of AlCoCrFeNiMoTiB_x_ HEA coating in 3.5 wt.% solution.

Sample	R_s_ (Ω·cm^2^)	Q_f_		R_f_ (kΩ·cm^2^)	Q_dl_		R_ct_ (kΩ·cm^2^)
		Y_o_ (10^−4^·Ω^−1^·cm^−2^·S^n^)	n_sl_		Y_o_ (10^−5^·Ω^−1^·cm^−2^·S^n^)	**n_sl_**	
S1	12.4	5.41	0.83	2.53	14.15	0.84	5.12
S2	14.1	4.17	0.87	3.12	8.52	0.86	7.64
S3	13.2	7.21	0.88	1.21	17.23	0.91	3.22
S4	12.7	8.92	0.86	0.67	25.62	0.81	2.78

## Data Availability

The original contributions presented in this study are included in the article. Further inquiries can be directed to the corresponding author.
